# Diagnostic Performance of Next-Generation Sequencing (NGS) in Indeterminate Thyroid Nodules: A Single Hospital Experience

**DOI:** 10.3390/ijms26094225

**Published:** 2025-04-29

**Authors:** Marco Capezzone, Maja Rossi, Sara Bardi, Eugenia Maria Morabito, Gilda Dalmazio, Giuseppe Iapichino, Simona Galassi, Serena Seralessandri, Liborio Torregrossa, Massimo Tosti Balducci, Elio Marchetti, Massimo Alessandri, Agostino Ognibene, Luigi De Napoli, Gabriele Materazzi, Silvia Cantara, Anello Marcello Poma

**Affiliations:** 1 UOSD of Endocrinology, Misericordia Hospital, 58100 Grosseto, Italy; marco.capezzone@uslsudest.toscana.it (M.C.); eugeniamaria.morabito@uslsudest.toscana.it (E.M.M.); gilda.dalmazio@uslsudest.toscana.it (G.D.); massimo.tostibalducci@uslsudest.toscana.it (M.T.B.); massimo2.alessandri@uslsudest.toscana.it (M.A.); 2 Laboratory Medicine Functional Area, Hospital Misericordia, 58100 Grosseto, Italy; maja.rossi@uslsudest.toscana.it (M.R.); sara.bardi@uslsudest.toscana.it (S.B.); giuseppe.iapichino@uslsudest.toscana.it (G.I.); simona.galassi@uslsudest.toscana.it (S.G.); serena.seralessandri@uslsudest.toscana.it (S.S.); agostino.ognibene@uslsudest.toscana.it (A.O.); 3 Department of Surgical Medical and Molecular Pathology, University Hospital of Pisa, 56124 Pisa, Italy; libo.torregrossa@gmail.com (L.T.); marcello.poma@med.unipi.it (A.M.P.); 4 Department of Pathology, Misericordia Hospital, 58100 Grosseto, Italy; elio.marchetti@uslsudest.toscana.it; 5 Division of Endocrine Surgery, Department of Surgical Pathology, University Hospital of Pisa, 56124 Pisa, Italy; l.denapoli@ao-pisa.toscana.it (L.D.N.); gabriele.materazzi@unipi.it (G.M.); 6 Department of Medical, Surgical and Neurological Sciences, 53100 Siena, Italy

**Keywords:** next-generation sequencing, indeterminate thyroid nodules, cytology

## Abstract

Fine-needle aspiration cytology (FNAC) is the gold standard to diagnose thyroid nodules but fails in discriminating the nature of indeterminate lesions. Molecular testing can improve the diagnosis of these nodules and next-generation sequencing (NGS) can be used to test many genes simultaneously. Assess the performance of an NGS 17-gene panel on thyroid indeterminate FNAC. One hundred five indeterminate FNACs, 30.5% high-risk (TIR3B) and 69.5% low-risk (TIR3A), were analyzed by NGS. For TIR3A, the rate of mutated samples was 10.9%, significantly lower (*p* = 0.0001) compared to TIR3B (63.6%). Twenty-two mutated and fourteen non-mutated samples were submitted to surgery. At histology, the overall malignancy was 85.7% in the indeterminate group that had a positive molecular test and 13.3% in the mutation-negative (*p* = 0.01). The 17-gene panel had a sensitivity of 90%, specificity of 87%, positive predictive value (PPV) of 91%, and negative predictive value (NPV) of 87%. We reported the utility of Ultrasound Malignancy Risk Stratification of Thyroid Nodules in Adults (EU-TIRADS) in selecting indeterminate nodules for molecular analysis, showing a significant correlation between US score and mutation (*p* = 0.004). The performance of a 17-gene panel based on NGS technology is promising, allowing the selection of indeterminate nodules to submit to surgery with a great specificity and PPV.

## 1. Introduction

Fine-needle aspiration cytology (FNAC) is considered the gold standard for diagnosing thyroid nodules (TNs), enabling the distinction between malignant and benign lesions in most cases. However, approximately 20% of FNAC samples yield an indeterminate diagnosis [1]. According to the Italian Consensus for the Classification and Reporting of Thyroid Cytology (ICCRTC), indeterminate TNs are cytologically classified as low-risk indeterminate (TIR3A), with an expected risk of malignancy of 5–15%, or as high-risk indeterminate (TIR3B), with an expected risk of malignancy of 15–30%. These classifications broadly correspond to “Atypia of undetermined significance or follicular lesion of undetermined significance (AUS/FLUS)” and “Follicular neoplasm or suspicious for a follicular neoplasm (FN/SFN)” of the Bethesda System for Reporting Thyroid Cytology (BSRTC), respectively [2,3].

Advances in identifying driver mutations in thyroid cancer (TC) have significantly improved the sensitivity of preoperative cancer detection through mutational panels. Several molecular tests categorized as either “rule out” or “rule in” tests have shown significant diagnostic improvement over FNAC [4,5,6,7]. A “rule out” test aims for a high sensitivity and negative predictive value (NPV) to effectively exclude malignancy, while a “rule in” test aims for a high specificity and positive predictive value (PPV) to indicate malignancy presence. Currently, molecular testing (including at least BRAFV600E, RET/PTC, and PAX8/PPARgamma rearrangements as well as RAS isoforms) is recommended by the 2015 American Thyroid Association (ATA) guidelines and the 2017 European Thyroid Association (ETA) guidelines as an adjunct technique to further stratify the risk of cytologically indeterminate nodules, potentially avoiding unnecessary diagnostic surgeries or identifying patients likely needing surgical treatment [1,8]. A larger vision of the 7-gene panel recommended by the ETA and ATA guidelines is represented by the ThyroSeq^®^ Genomic Classifier (GC) test, which utilizes next-generation sequencing to analyze the DNA and RNA of 112 thyroid-related genes for four main classes of molecular alterations, including mutations, gene fusions, copy number alterations, and gene expression alterations. However, panels like this, containing numerous markers, are not widely used in Europe due to their high cost and lack of reimbursement by national healthcare systems, limiting their application in routine clinical practice [9]. To address this limitation, some authors have reported data on more limited, customized “rule-in” panels that detect the most common genetic alterations in thyroid cancer, albeit with a lower sensitivity compared to next-generation sequencing (NGS) and comprehensive gene expression profile panels.

In this study, we present our experience using a 17-gene panel based on next-generation sequencing (NGS) technology, performed on indeterminate thyroid FNAC samples from a single Italian center. Compared to the other proposed solutions, this test has the advantage of being a cost-effective CE-IVD kit representing an improvement if compared with “in house” PCR or larger panels not covered by the health system.

## 2. Results

### 2.1. Nodule Characteristics

A total of 105 indeterminate FNAC samples, 32 (30.5%) TIR3B and 73 (69.5%) TIR3A, derived from 104 patients (72 females and 32 males) were analyzed by NGS. The age of patients ranged from 22 to 82 years (median 59 years) and the median nodule size was 21 mm. According to the EU-TIRADS classification, there were 33 (31.4%) nodules scored EU-TIRADS 2, 48 (45.8%) EU-TIRADS 3, 21 (20%) EU-TIRADS 4, and 3 (2.8%) EU-TIRADS 5. The characteristics of patients (n = 104) and of the indeterminate thyroid nodules (n = 105), according to cytological category, are reported in Table 1.

### 2.2. Molecular Test Performance

**Figure 1:**
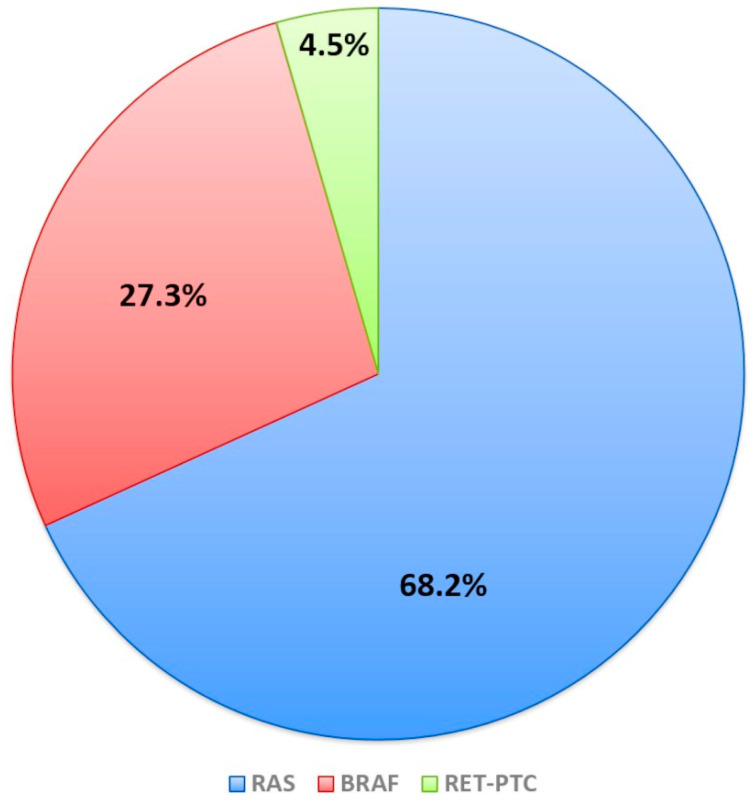
Prevalence of mutations among mutated nodules.

The presence of pathological/likely pathological mutations by NGS testing was found in 22/105 (21%) FNAC samples. Considering only the 22 mutated nodules, RAS genes point mutations (2 HRAS, 2 KRAS, and 11 NRAS) were present in 15 of 22 (68.2%), BRAF point mutations in 6 (27.3%), and RET/PTC rearrangements in 1 of 22 (4.5%) mutated samples (Figure 1).

**Figure 2:**
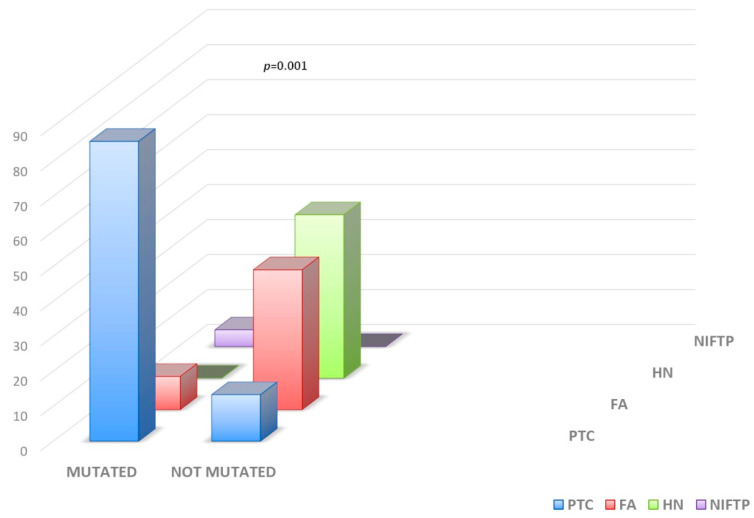
Prevalence of mutations in relation to histology. FA, follicular adenoma; HN, hyperplastic nodule; NIFTP, non-invasive follicular thyroid neoplasm with papillary-like nuclear features; PTC, papillary thyroid carcinoma.

All but one BRAF mutations were p.V600E; the only exception was a c.1799_1812delinsAT, p.V600_W604delinsD mutation. In the TIR3A group, the rate of mutated samples was 10.9% (8/73), significantly lower (*p* = 0.0001) compared to that of the TIR3B group (14/32, 63.6%). In particular, in the TIR3A group, we observed the presence of RAS mutations in 7/8 (87.5%) and BRAF mutation in 1/8 (22.5%) mutated samples, and in the TIR3B group, we found 8/14 (57.1%) RAS mutations, 4/14 (28.5%) BRAF mutations, and 1/14 (7.1%) RET/PTC rearrangement (Table 1). A total of 36/104 (34.3%) patients were submitted to thyroid surgery. The surgical group included 22 NGS mutated nodules belonging to 21 patients (1 patient carrying RAS mutation refused surgery) and 14 thyroid nodules belonging to 15 patients negative for any mutation. The overall malignancy rate at surgery was 18/21 (85.7%) in the indeterminate group that had positive molecular testing, and 2/15 (13.3%) in the mutation-negative nodule group (*p* = 0.01). Among 21 FNAC nodules that were mutation-positive by NGS analysis, 18/21 samples were confirmed as histologically malignant (all papillary thyroid cancers, PTCs), 1/21 as non-invasive follicular thyroid neoplasm with papillary-like nuclear features (NIFTP), and 2/21 as follicular adenomas (FAs). Among 15 mutation-negative FNA samples, there were 7/15 hyperplastic nodules (HNs), 6/15 FAs, and 2/15 PTCs (Figure 2).

**Figure 3:**
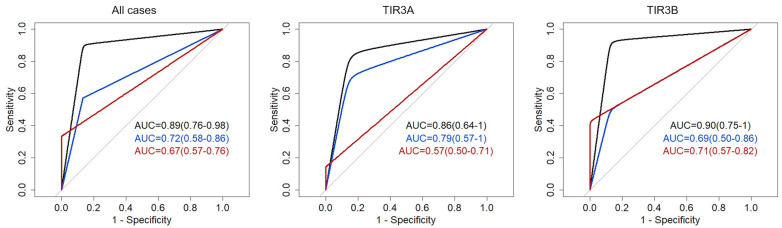
Diagnostic performance of the 17-gene NGS panel. ROC curves were built for all cases (**left**), TIR3A cases only (**center**), and TIR3B cases only (**right**). In each panel, AUC of all mutations (black), RAS-like mutations (blue), and BRAF-like mutations (red) are shown.

The performance of the 17-gene panel including the sensitivity, specificity, accuracy, positive predictive value (PPV), and negative predictive value (NPV) is reported in Table 2 and Figure 3. Considering all mutations (both BRAF- and RAS-like) and all nodules, the 17-gene panel had an AUC of 89%, sensitivity of 90%, specificity of 87%, PPV of 91%, and NPV of 87%. In the TIR3A-group, the PPV and NPV decreased to 86%, respectively, while in the TIR3B-group, the NPV was 87% and the PPV increased to 93%. Evaluating the diagnostic performance of the NGS panel test in relation to the presence of RAS- and BRAF-like mutations, we observed that, considering all cases, in patients with RAS-like mutations, the specificity was 87%, sensitivity 57%, NPV 59%, and PPV of 87% while in the BRAF-like group, the specificity and PPV were 100%, sensitivity 33%, and NPV 52%. In the TIR3A group, the presence of RAS-like mutations determined a specificity of 86%, sensitivity of 71%, NPV of 75%, and PPV of 86% whereas the presence of BRAF-like mutations determined a specificity and PPV of 100%, sensitivity of 14%, and NPV of 54%. In the TIR3B group, the presence of RAS-like mutations determined a specificity of 87%, sensitivity of 50%, NPV of 50%, and PPV of 89% whereas the presence of BRAF-like mutations determined a specificity and PPV of 100%, sensitivity of 43%, and NPV of 50%.

**Figure 4:**
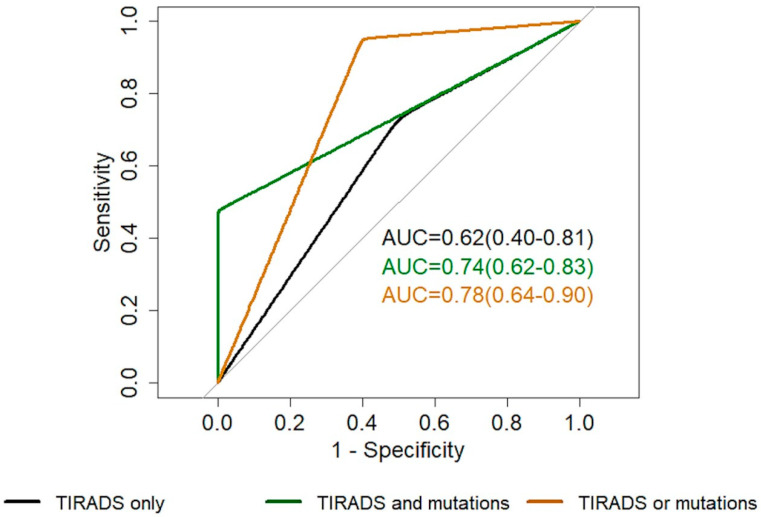
Diagnostic performance of EU-TIRADS risk class alone (EU-TIRADS 2–3 vs. 4–5, black) and combined with molecular test. In detail, in green is the ROC curve considering as a predictor of malignancy both EU-TIRADS 4–5 and the presence of mutations; in orange is the ROC curve considering as a predictor of malignancy EU-TIRADS 4–5 or the presence of mutations.

We reported the utility of the EU-TIRADS ultrasound score in selecting indeterminate nodules for molecular analysis showing a significant correlation between the US score and mutational status (*p* = 0.004), suggesting that US patterns are significantly associated with mutational status. The frequency of nodules with a mutation increases alongside the higher US risk class levels (14.8% in EU-TIRADS 2–3, 41.7% in EU-TIRADS 4–5; *p* = 0.004). The performance of EU-TIRADS alone and combined with the presence of the mutation is reported in Table 3 and Figure 4.

## 3. Discussion

The diagnosis of indeterminate thyroid nodules is challenging as up to 30% of the cases are classified as indeterminate [10]. International and national guidelines recommend molecular testing, whenever possible, to refine the risk of malignancy of indeterminate nodules [8,11,12,13]. Several molecular tests have been developed to improve the diagnosis of indeterminate lesions, but many of them have high costs and are not commonly used in Europe. To overcome this limitation, customized panels have been set up. These panels can detect the most frequent genetic alterations in thyroid cancer [14,15,16,17]. Integration of the results of molecular testing with cytology and US risk category could allow the stratification of patients according to their risk of malignancy, enabling possible refinement of their treatment options [18,19,20,21]. In this study, we evaluated the diagnostic performance of a 17-gene panel based on NGS technology, performed on a series of consecutive thyroid indeterminate FNAC samples, showing that NGS on FNAC is feasible with good sequencing quality. Our panel showed an overall sensitivity of 90% with an NPV of 87% in a series of 105 nodules classified as low-risk indeterminate (TIR3A) and as high-risk indeterminate (TIR3B), demonstrating that the diagnostic accuracy of FNA cytology can be increased by applying this 17-gene NGS panel in the clinical routine. The most frequently found mutations were in the RAS genes as expected, followed by BRAF and RET-PTC. In addition, the prevalence of mutations in high-risk lesions (TIR3B) was significantly higher than in low-risk nodules (TIR3A). Among the 35 nodules submitted to surgery, only 2 yielded a mutation-negative NGS test and resulting, at histology, in being malignant. On the other hand, two benign lesions, namely two FAs, harbored RAS gene mutations.

Notably, in our series, RAS-like mutations showed a good overall specificity and PPV, especially among high-risk indeterminate lesions. These findings are in line with other reports in which the presence of RAS mutation was strongly suggestive of malignancy or lesions deserving surgery [2,22,23]. On the other hand, some authors have shown that RAS-mutant nodules with indeterminate cytology are clinically low risk and might be followed with active surveillance [24]. While it is generally accepted that the finding of an RAS mutation increases the preoperative risk of malignancy, the clinical management of RAS-mutant nodules with indeterminate cytology remains a highly debated topic [25,26].

Our findings also show that the integration of the molecular test with EU-TIRADS risk class might provide useful information. Indeed, the coexistence of oncogene mutations with EU-TIRADS 4 or 5 classes provides a specificity and PPV of 100%. On the other hand, using the presence of gene mutations or US high risk (i.e., EU-TIRADS 4 and 5) as predictors of malignancy, we obtained a sensitivity of 95% and an NPV of 90%. The above considerations were obtained irrespective of the cytology diagnosis (i.e., TIR3A or TIR3B).

Regarding technical aspects, NGS requires less starting material (DNA or RNA) than that needed for traditional methods and several studies reported its superiority in terms of sensitivity and cost optimization compared to traditional methods [27,28,29]. A major advantage of NGS is its ability to screen multiple mutations in multiple genes simultaneously without the need to perform several sequential tests. A recent survey carried out in the Netherlands demonstrated that performing in-house NGS can save up to 2 million euros annually in the management of patients with indeterminate FNA [30]. Our results also demonstrated the possibility of identifying novel molecular alterations using NGS panels. In fact, we detected a non-V600E mutation, c.1799_1812delinsAT, p.V600_W604delinsD, with no similar findings yet reported in the literature [31].

An important observation is that in our study group, there is a high prevalence of malignancy in the TIR3A group. This prevalence is higher than that usually reported in the guidelines, but it is still in line with the results of other papers [2].

## 4. Materials and Methods

### 4.1. Study Population

Eligible patients had a thyroid nodule >10 mm with indeterminate cytology, processed with the liquid-based cytology ThinPrep5000™ method (Hologic Co., Marlborough, MA, USA), according to the Italian Consensus for the Classification and Reporting of Thyroid Cytology (ICCRTC) in the six months before inclusion [2]. We prospectively collected 105 consecutive indeterminate thyroid nodules belonging to 104 patients followed at the Section of Endocrinology of the Misericordia hospital (Grosseto, Italy) between March 2023 and June 2024. All thyroid nodules were evaluated using a high-resolution ultrasound color Doppler apparatus with a 12 MHz linear transducer. The ultrasound reports were classified according to European Thyroid Association Guidelines for Ultrasound Malignancy Risk Stratification of Thyroid Nodules in Adults (EU-TIRADS) [32]. Thyroid nodules were divided into four groups: EU-TIRADS 2 (benign category); EU-TIRADS 3 (low-risk category); EU-TIRADS 4 (intermediate-risk category); EU-TIRADS 5 (high-risk category). All FNA were performed under ultrasound guidance using a 23 g needle depending on institutional practice. The study protocol provided for two dedicated passes of FNA washed in a tube containing nucleic acid preservative solution (RNA protect, Qiagen, Hilden, Germany). Thyroid nodules were cytologically defined as low-risk indeterminate (TIR3A) or as high-risk indeterminate (TIR3B) according to the ICCRTC. Written informed consent was obtained from all patients. The procedure of FNAC associated with NGS was approved as a routine clinical activity in Misericordia hospital, according to diagnostic protocol AMEL number 002 of 26 March 2024, Azienda USL Toscana Sud-Est.

### 4.2. DNA and RNA Extraction

Material obtained from fine-needle aspiration was added to 300 µL of RNA protect Cell Reagent (Qiagen) and stored at 4 °C for a maximum of one week. At the time of extraction, 150 µL was used for DNA extraction with the QIAamp DNA Blood Mini kit (Qiagen) and the remaining material was used for RNA extraction with RNeasy Mini kit (Qiagen) in accordance with the manufacturer’s instructions. Before the addition of the lysis reagent, for both extractions, samples were centrifuged at 5000× *g* × 5 min to allow cells to pellet. Both DNA and RNA were evaluated by Nanodrop (Thermofisher, Waltham, MA, USA) in terms of quantity and quality by evaluating the ratio Ab260/Abs280. RNA was then stored at −80 °C and DNA at −20 °C if not immediately processed.

### 4.3. Myriapod NGS Cancer Panel DNA and RNA Analysis

DNA previously extracted was analyzed by the Myriapod NGS Cancer panel DNA (Diatech Pharmacogenetics, Jesi, Italy) on the iSeq platform (Illumina, San Diego, CA, USA). The input material was 10 ng/reactions. This panel allows the investigation of 123 DNA regions involving the hot-spots of 17 distinctive cancer-related genes (ALK, BRAF, EGFR, ERBB2, FGFR3, HRAS, IDH1, IDH2, KIT, KRAS, MET, NRAS, PDGFRA, PIK3CA, POLE, RET, and ROS1). Coverage and variant calling data analysis were carried out by Myriapod NGS Data analysis Software v 2.0 (Diatech Pharmacogenetics, Jesi, Italy). The procedure sensitivity was around 5%. In detail, samples with a minimum coverage of 500X and a variant allele frequency ≥5% were selected. The IGV software v.2.2.7 (Broad Institute and the Regents, University of California, CA, USA) was used to manually check the variants called. Finally, mutational frequency was recorded.

RNA libraries were generated using the Myriapod^®^ NGS Cancer panel RNA (Diatech Pharmacogenetics) according to the manufacturer’s instructions. In detail, RNA was backtranscribed into cDNA using random hexamers. Then, cDNA was amplified by multiplex PCR using two mixtures of primers to obtain fragments, between 47 and 184 bases in length, including the fusions of interest and endogenous control genes.

The RNA panel allows the detection of fusions regarding 10 cancer genes: ALK, ROS1, RET, NTRK1, NTRK2, NTRK3, FGFR2, FGFR3, PPARG, and the skipping of exon 14 of MET. Sequencing data analysis was carried out on Myriapod^®^ NGS Data Analysis Software v. 2.0 (Diatech Pharmacogenetics, Jesy, Italy). The fusion partners of the genes present in the panel are listed in detail in Supplementary Table S1. The analytical sensitivity of the kit was 5 ng/reaction. The molecular assessment cut-off was >5000 reads on target; the number of reads associated with specific fusions was >30; and the number of reads associated with non-specific fusions was >500.

### 4.4. Statistical Analysis

Differences between categorical variables were assessed by the Fisher’s exact text. The diagnostic performance of molecular testing was computed by Receiver Operator Characteristics (ROC) analysis. Area under the curve (AUC), sensitivity, specificity, accuracy, negative predictive value (NPV), and positive predictive value (PPV) were calculated. The 95% confidence intervals were computed by 2000 bootstrap resampling. The analyses were performed in R environment (v.4.3.3, https://www.r-project.org/, last accessed 7 March 2025).

## 5. Conclusions

In conclusion, the possibility to perform NGS on indeterminate thyroid nodules allows us to discriminate with a greater specificity and PPV which nodules should be subject to surgery. The results of the study demonstrate that the 17-gene NGS panel also provides high sensitivity for cancer detection in thyroid nodules with indeterminate cytology, which should allow improved management for these patients.

## Figures and Tables

**Table 1:** Baseline characteristics of patients (n = 104) and thyroid nodules (n = 105) according to cytological diagnosis.

Parameters	TIR3A (N = 73)	TIR3B (N = 32)	*p*
Age at Diagnosis (Years)			0.5
Mean ± SD	57.5 ± 12.1	58.9 ± 13.2	
Sex			0.2
Males	20 (27.4)	13 (40.6)	
Females	53 (72.6)	19 (59.4)	
EU-TIRADS: N (%)			0.9
2	23 (31.5)	10 (31.2)	
3	33 (45.2)	15 (46.9)	
4	15 (20.5)	6 (18.7)	
5	2 (2.8)	1 (3.2)	
Size of Thyroid Nodule (mm)			0.1
Mean ± SD	22.3 ± 9.1	25.6 ± 10.1	
Mutations: N (%)			0.0001
*BRAF*	1 (1.4)	5 (15.7)	
*NRAS*	5 (6.9)	6 (18.7)	
*KRAS*	2 (2.7)	0	
*HRAS*	0	2 (6.3)	
*RET-PTC1*	0	1 (3.1)	
No Mutations	65 (89)	18 (56.2)	

**Table 2:** Performance of the ampliSeq 17-NGS panel in thyroid nodules with indeterminate cytology.

	All Mutations	Ras-like Mutations	Braf-like Mutations
Total Cases			
AUC	0.89 (0.76–0.98)	0.72 (0.58–0.86)	0.67 (0.57–0.76)
Specificity	0.87 (0.67–1)	0.87 (0.67–1)	1 (1–1)
Sensitivity	0.90 (0.76–1)	0.57 (0.38–0.81)	0.33 (0.14–0.52)
Accuracy	0.89 (0.78–0.97)	0.69 (0.56–0.83)	0.61 (0.50–0.72)
NPV	0.87 (0.72–1)	0.59 (0.48–0.75)	0.52 (0.45–0.60)
PPV	0.91 (0.79–1)	0.87 (0.70–1)	1 (1–1)
TIR3A Cases			
AUC	0.86 (0.64–1)	0.79 (0.57–1)	0.57 (0.50–0.71)
Specificity	0.86 (0.57–1)	0.86 (0.57–1)	1 (1–1)
Sensitivity	0.86 (0.57–1)	0.71 (0.43–1)	0.14 (0–1)
Accuracy	0.86 (0.64–1)	0.79 (0.57–1)	0.57 (0.50–0.71)
NPV	0.86 (0.63–1)	0.75 (0.56–1)	0.54 (0.50–0.64)
PPV	0.86 (0.64–1)	0.86 (0.60–1)	1 (1–1)
TIR3B Cases			
AUC	0.90 (0.75–1)	0.69 (0.50–0.86)	0.71 (0.57–0.82)
Specificity	0.87 (0.62–1)	0.87 (0.62–1)	1 (1–1)
Sensitivity	0.93 (0.79–1)	0.50 (0.51–0.79)	0.43 (0.14–0.71)
Accuracy	0.91 (0.77–1)	0.63 (0.45–0.82)	0.64 (0.45–0.82)
NPV	0.87 (0.67–1)	0.50 (0.37–0.67)	0.50 (0.40–0.67)
PPV	0.93 (0.81–1)	0.89 (0.67–1)	1 (1–1)

**Table 3:** Performance of EU-TIRADS US score alone and combined with the presence of thyroid oncogenes mutations.

	EU-TIRADS Alone (2–3 vs. 4–5)	EU-TIRADS 4–5 and Presence of Mutations	EU-TIRADS 4–5 or Presence of Mutations
AUC	0.62 (0.40–0.81)	0.74 (0.62–0.83)	0.78 (0.64–0.90)
Specificity	0.50 (0–1)	1 (1–1)	0.60 (0.33–0.80)
Sensitivity	0.73 (0–1)	0.48 (0.29–0.67)	0.95 (0.86–1)
Accuracy	0.65 (0.35–0.83)	0.69 (0.58–0.81)	0.81 (0.69–0.92)
NPV	0.50 (0.35–0.80)	0.58 (0.50–0.68)	0.90 (0.70–1)
PPV	0.74 (0.65–0.91)	1 (1–1)	0.77 (0.67–0.87)

## Data Availability

The original contributions presented in this study are included in the article. Further inquiries can be directed to the corresponding author.

## Supplementary Materials

The following supporting information can be downloaded at: https://www.mdpi.com/article/10.3390/ijms26094225/s1.
